# Symptoms and delay times during myocardial infarction in 694 patients with and without diabetes; an explorative cross-sectional study

**DOI:** 10.1186/s12872-016-0282-7

**Published:** 2016-05-26

**Authors:** Karin Hellström Ängerud, Ingela Thylén, Sofia Sederholm Lawesson, Mats Eliasson, Ulf Näslund, Christine Brulin, on behalf of the SymTime Study Group

**Affiliations:** Cardiology, Heart Centre and Department of Nursing, Umeå University, Umeå, Sweden; Department of Cardiology and Department of Medical and Health Sciences, Linköping University, Linköping, Sweden; Department of Public Health and Clinical Medicine, Sunderby Research Unit, Umeå University, Umeå, Sweden; Department of Public Health and Clinical Medicine, Cardiology, Heart Centre, Umeå University, Umeå, Sweden; Department of Nursing, Umeå University, Umeå, Sweden

**Keywords:** Myocardial infarction, Diabetes mellitus, Symptoms, Patient delay

## Abstract

**Background:**

In myocardial infarction (MI) a short pre-hospital delay, prompt diagnosis and timely reperfusion treatment can improve the prognosis. Despite the importance of timely care seeking, many patients with MI symptoms delay seeking medical care. Previous research is inconclusive about differences in symptom presentation and pre-hospital delay between patients with and without diabetes during MI. The aim of this study was to describe symptoms and patient delay during MI in patients with and without diabetes.

**Methods:**

Swedish cross-sectional multicentre survey study enrolling MI patients in 5 centres within 24 h from admittance.

**Results:**

Chest pain was common in patients both with and without diabetes and did not differ after adjustment for age and sex. Patients with diabetes had higher risk for shoulder pain/discomfort, shortness of breath, and tiredness, but lower risk for cold sweat. The three most common symptoms reported by patients with diabetes were chest pain, pain in arms/hands and tiredness. In patients without diabetes the most common symptoms were chest pain, cold sweat and pain in arms/hands. Median patient delay time was 2 h, 24 min for patients with diabetes and 1 h, 15 min for patients without diabetes (*p* = 0.024).

**Conclusion:**

Chest pain was common both in patients with and without diabetes. There were more similarities than differences in MI symptoms between patients with and without diabetes but patients with diabetes had considerably longer delay. This knowledge is important not only for health care personnel meeting patients with suspected MI, but also for the education of people with diabetes.

## Background

A short pre-hospital delay, leading to prompt diagnosis and treatment of patients with a myocardial infarction (MI), can reduce mortality, improve prognosis, and shorten the hospital stay [1–3]. Despite the importance of timely care seeking, many patients delay their first medical contact (FMC) [4], leading to increased morbidity and mortality because treatment is not given in a timely manner [3]. Pre-hospital delay can be divided into three phases: 1) patient decision time, from symptom onset to the decision to seek medical care; 2) the time from the decision to FMC; and 3) the time from FMC to hospital arrival, including the transportation time. Transportation to the hospital contributes only a little to pre-hospital delay; the patient’s decision time constitutes the greatest part of total pre-hospital delay [5].

Findings based on in-depth interviews show that the interpretation of symptoms and the decision to seek medical care during an MI are multifaceted and complex [6–9]. Symptoms are key factors in the representation of a health threat [10] and the experience and interpretation of symptoms are important factors in deciding how to act and respond to signs and symptoms. Misinterpreting symptoms or not taking them seriously have been described associated with longer patient delay [6, 9, 11]. A previous literature review found that socio-demographic, clinical, cognitive, psychological, behavioural, and contextual factors influence patient delay and are important in understanding the phenomenon of delay [12]. Older age [13, 14], female sex [4, 14], and comorbidities [13, 15] have been found to be associated with prolonged delays according to previous research.

Previous research is inconclusive on whether there are differences between patients with and without diabetes regarding symptoms and pre-hospital delays times. Some studies have demonstrated that patients with diabetes less frequently report chest pain [16] and have longer pre-hospital delays [17],while others have found no such differences [15, 18, 19]. Because diabetes is associated with an increased risk of developing MI [20] and a lower post-MI survival rate [21–23], it is crucial that patients with diabetes recognize possible MI symptoms and seek care promptly. Better understanding of symptom presentation and patient delay times in patients with diabetes is needed to improve time to treatment. Therefore, the aim of our study was to describe symptoms and patient delay during MI in patients with and without diabetes.

## Methods

This study is a part of a Swedish multicentre survey study, SymTime, and had a descriptive and comparative cross-sectional design. The study was performed between November 2012 and January 2014 with participants from five hospitals in northern and southeast Sweden.

### Participants

The study population consisted of 694 patients hospitalized with MI. Patients were eligible if they (i) had a diagnosis of ST-elevation MI (STEMI) or non-ST-elevation MI (NSTEMI) according to the European Society of Cardiology guidelines [3, 24], (ii) were willing to participate, and (iii) were able to fill in the questionnaire by themselves or with help from hospital personnel or family. Participants were enrolled in the study within 24 h of their admission to the coronary care unit and should be clinically stable at the time point of inclusion.

### Data collection

A previously validated self-administered questionnaire was used to access self-reported data on symptoms, pre-hospital delay, and initial response to symptoms in MI patients. This questionnaire was originally developed and tested a decade ago in a Swedish chest pain population (i.e., acute MI, angina pectoris, and/or non-cardiac chest pain) [19]. Prior to the present study, a new review of the literature and expert validation of the questionnaire were conducted in collaboration with the original developer. This validation procedure is described previously [25]. The modified questionnaire included 35 items covering four domains; (i) background characteristics (sex, age, marital status, medical history, distance to nearest hospital, and educational level), (ii) symptoms (how patients experienced and attributed their symptoms, and how they rated their pain or discomfort on a numeric rating scale (NRS) of 0–10, (iii) course of events (e.g., actions taken after symptom onset; whether or not they were alone; who they contacted first; whether they practiced any self-care); and finally, (iv) mode of transport to hospital, including time point measurements based on patients’ statements.

The modified questionnaire was administered to the patients by the staff nurse in charge or the nurse responsible for the study at each hospital. The patients were instructed to choose the alternative (s) that best reflected their pre-hospital experiences. Data about diabetes, hypertension, heart failure, previous MI, previous angina, and smoking habits were self-reported (from options on the questionnaire). Patients with diabetes included both patients with diabetes type 1 and type 2. In addition, information about comorbidities, certain time point measurements, and FMC was registered by the nurse responsible for the patient. Patient delay time was defined as the time interval between onset of symptoms and FMC. FMC was defined as the first contact the patient made with any of emergency medical service (EMS), Primary Healthcare, Swedish Healthcare Direct (a joint service number, 1177, staffed by advisory nurses 24/7) or emergency room.

### Statistical methods

Characteristics of the participants were described using frequencies, proportions, means (m), and standard deviations (sd) and medians (Md) and quartiles (Q1, Q3). Comparisons between groups were made using chi-square test, Fisher’s exact test, Mann-Whitney *U* test, and Student *t*-test as appropriate. Multiple logistic regression analyses were used to adjust for age and sex. Delays in patients with and without diabetes were compared non-parametrically with Mann-Whitney *U* test as the distribution was non-normal. We dichotomised patient delay time using < 2 h/≥ 2 h as cut-of, and chi-square test was used to test for difference between groups in univariate analyses. To adjust for age and sex multiple logistic regression was used. A *p*-value < 0.05 was considered significant. The statistical analyses were performed using SPSS version 22.0 for Windows.

### Ethical aspects

Patients were hemodynamically stable and pain free when they were informed about the study and asked to participate. A written consent was obtained from the patients before inclusion in the study. This study was approved by the regional Ethical Review Board, Linköping, Sweden (D-nr 2012/201-31, 2012/338-32), and conformed with the Declaration of Helsinki [26].

## Results

Of the 694 participants, 96 (13.8 %) had diabetes. The mean age was 66.5 (sd 12.1) in patients with diabetes and 65.9 (sd 11.2) in patients without. Patients with diabetes had a significantly higher prevalence of hypertension and angina pectoris. The diabetes group included a higher proportion of women (*p* = 0.051) and individuals with previous MI (*p* = 0.055) than the non-diabetes group. Other background characteristics were quite similar between the groups (Table 1).

**Table 1:** Background characteristics of the participants

Background characteristics	Patients with diabetes, *n* = 96	Patients without diabetes, *n* = 598	*p*-value
**Age**, **mean (sd)**	66.5 (12.1)	65.9 (11.2)	0.6
**Sex**, **male**, ***n*** **(%)**	65 (67.7)	460 (76.9)	0.051
**Distance to hospital**, ***n*** **(%)** ^**a**^			
≤1 km	8 (8.3)	35 (6.0)	0.6
1**–**10 km	32 (33.3)	208 (35.4)	
10**–**50 km	38 (39.6)	254 (43.2)	
> 50 km	18 (18.8)	91 (15.5)	
**Marital status**, ***n*** **(%)**			
Married/cohabitant	66 (68.8)	429 (71.7)	0.5
**Educational level**, ***n*** **(%)** ^**a**^			
Compulsory school	48 (50.0)	253 (42.4)	0.4
Gymnasium	31 (32.3)	221 (37.1)	
Higher education	17 (17.7)	122 (20.5)	
**History of**, ***n*** **(%)** ^**a**^			
Hypertension	80 (83.3)	249 (42.1)	**<0.001**
Angina pectoris	25 (27.8)	78 (13.3)	**<0.001**
Atrial fibrillation	7 (7.8)	32 (5.5)	0.4
Heart failure	5 (5.5)	17 (2.9)	0.2
MI	20 (22.0)	84 (14.2)	0.055
Stroke	5 (5.2)	21 (3.5)	0.4
Current smoker	18 (18.8)	132 (22.3)	0.4
**Type of infarction**, ***n*** **(%)** ^**a**^			
STEMI	74 (77.1)	458 (76.6)	0.9
NSTEMI	22 (22.9)	140 (23.4)	

^a^Differences in percentages are due to missing responses

Significant results (*p* < 0.05) are presented in bold style

### Symptoms and symptom interpretations

Chest/thoracic pain, discomfort, or pressure was the most common symptom reported among patients with and without diabetes. In the univariate analyses, patients with diabetes reported chest/thoracic pain, discomfort, or pressure significantly less often than patients without diabetes (81.3 % vs 89.5 %, *p* = 0.020). After adjusting for age and sex, this difference did not reach significance (*p* = 0.065) (Table 2). The three most common symptoms reported by patients with diabetes were chest pain, pain in arms/hands and tiredness. In patients without diabetes the most common symptoms were chest pain, cold sweat and pain in arms/hands. Even after adjustment for age and sex, patients with diabetes had higher risk for shoulder pain/discomfort, shortness of breath, and tiredness but lower risk for cold sweats. There were no significant between-group differences in other symptoms (Table 2).

**Table 2:** Symptom presentation in patients with and without diabetes. Crude and adjusted odds ratio (OR) and confidence interval (CI) for having different symptoms

Symptoms	Patients with diabetes, *n* = 96	Patients without diabetes, *n* = 598		
**Pain, discomfort, or pressure location**	*n* (%)	*n* (%)	Crude OR (95 % CI)	Adjusted OR (95 % CI)
Chest or thoracic	78 (81.3)	535 (89.5)	**0.51 (0.29–0.91)**	0.57 (0.31–1.04)
Throat or neck	25 (26.0)	118 (19.7)	1.43 (0.87–2.36)	1.36 (0.82–2.25)
Jaw or teeth	15 (15.6)	65 (10,9)	1.52 (0.83–2.79)	1.44 (0.78–2.67)
Back	18 (18.8)	99 (16.6)	1.16 (0.67–2.03)	1.07 (0.60–1.88)
Stomach	10 (10.4)	48 (8.0)	1.33 (0.65–2.73)	1.36 (0.66–2.80)
Shoulders	34 (35.4)	110 (18.4)	**2.43 (1.53–3.88)**	**2.30 (1.43–3.70)**
Arms/hands	50 (52.1)	331 (55.4)	0.88 (0.57–1.35)	0.84 (0.54–1.30)
**Other symptoms**				
Numbness in arms/hands	24 (25.0)	183 (30.6)	0.76 (0.46–1.24)	0.76 (0.46–1.25)
Tiredness	41 (42.7)	186 (31.1)	**1.65 (1.06–2.56)**	**1.62 (1.03–2.52)**
Weakness	37 (38.5)	234 (39.1)	0.98 (0.63–1.52)	0.96 (0.61–1.49)
Shortness of breath	40 (41.7)	184 (30.8)	**1.61 (1.03–2.50)**	**1.61 (1.03–2.51)**
Vertigo/pre-syncope	23 (24.0)	140 (23.4)	1.03 (0.62–1.71)	1.02 (0.61–1.69)
Nausea/vomiting	26 (27.1)	181 (30.3)	0.86 (0.53–1.39)	0.78 (0.47–1.28)
Cold sweat	40 (41.7)	333 (55.7)	**0.57 (0.37–0.88)**	**0.56 (0.36–0.86)**
Anxiety	12 (12.5)	79 (13.2)	0.94 (0.49–1.80)	0.92 (0.48–1.77)
Fear	18 (18.8)	132 (22.1)	0.82 (0.47–1.41)	0.77 (0.44–1.34)
General sick feeling	16 (16.7)	88 (14.7)	1.16 (0.65–2.08)	1.09 (0.61–1.97)
Other	9 (9.4)	41 (6.9)	1.40 (0.66–2.99)	1.37 (0.64–2.94)

Significant results are presented in bold style. Adjusted OR: adjusted for age and sex

Of the 18 possible symptoms presented in the questionnaire, there was no difference in number of reported symptoms between patients with and without diabetes, median 5.0 (*p* = 0.600). There was also no difference in pain intensity between patients with and without diabetes, median 7 on NRS. More than half of the participants in both groups described the pain as “persistent”, followed by “come and go” or “increasing”, and only a few described the pain as “transient”. There were no significant differences between the groups in the descriptions of pain continuity (Table 3).

**Table 3:** Symptom characteristics and symptom interpretation in patients with and without diabetes

	Patients with diabetes, *n* = 96	Patients without diabetes, *n* = 598	*p*-value
How the pain occurred, *n* (%)			
-Transient	5 (5.4)	17 (2.9)	0.7
-Come and go	23 (24.7)	145 (24.9)	
-Persistent	48 (51.6)	317 (54.4)	
-Increasing	17 (18.3)	104 (17.8)	
Interpretation of symptoms as originating from the heart, *n* (%)	64 (66.7)	398 (66.7)	1.0
Number of symptoms >5, *n* (%)	43 (44.8)	223 (37.3)	0.2
Number of symptoms, Md [Q_1_,Q_3_]	5.0 [3.0,7.0]	5.0 [3.0,6.0]	0.6
Pain intensity, NRS, Md [Q_1_,Q_3_]	7.0 [6.0,8.0]	7.0 [6.0,8.0]	0.9

*NRS* numeric rating scale of 0–10

About two thirds of the participants in both groups reported interpreting the symptoms as originating from the heart (Table 3). The second and third most common attributions of the origin of the symptoms were the stomach (30.6 % in patients with diabetes vs. 35.5 % in patients without diabetes) and the muscles (30.6 % in patients with diabetes vs 28.1 % in patients without diabetes), with no significant differences between the groups. Of those who did not interpret the symptoms as originating from the heart, more patients with diabetes than without were “entirely sure the symptoms did not originate from the heart” (62.9 % vs. 40.5 %, *p* = 0.013).

### Patient delay time

**Fig. 1:**
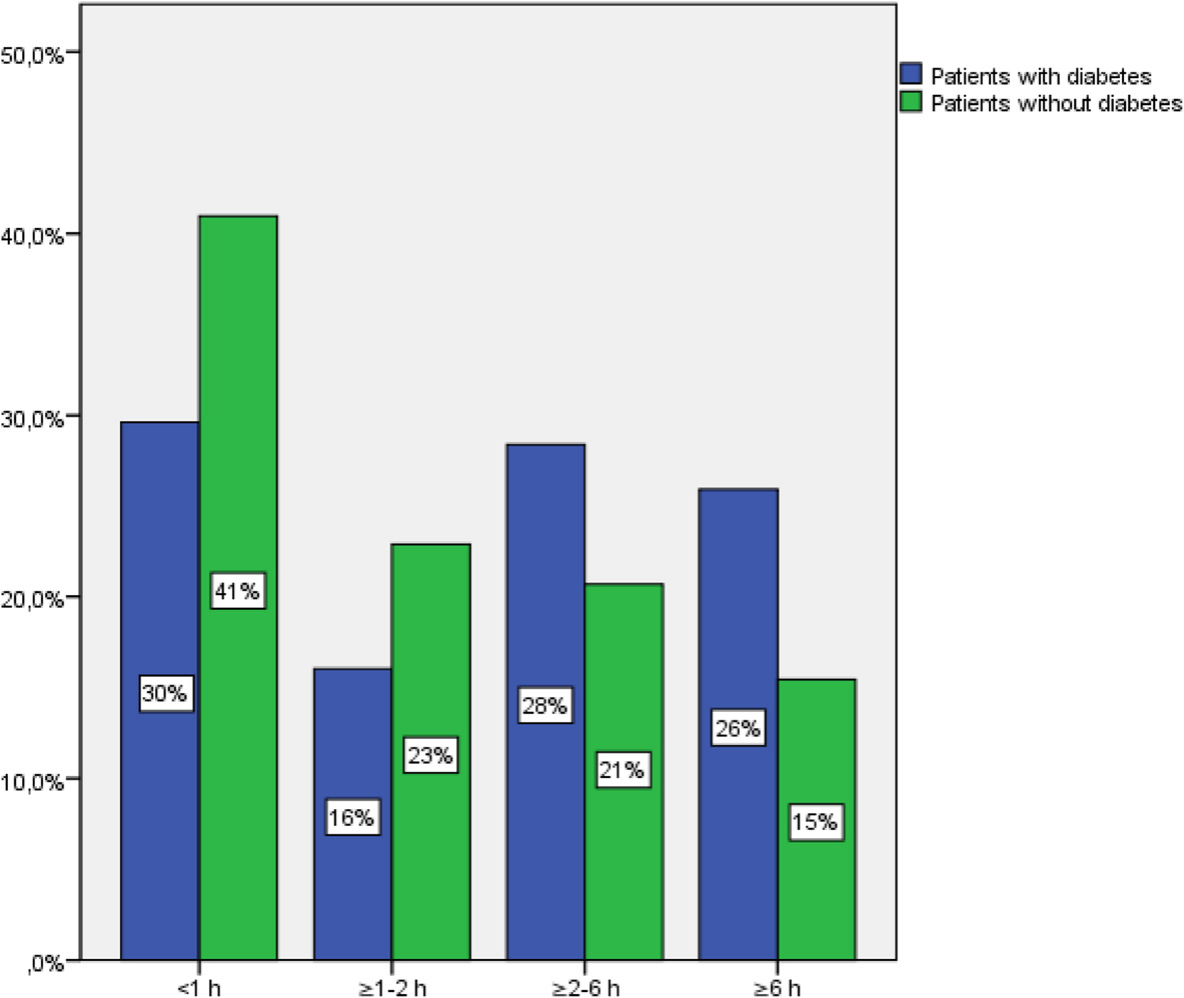
Distribution of time from symptom onset to first medical contact in patients with and without diabetes

Median patient delay time from symptom onset to FMC was 2 h, 24 min (Q_1,_ 0:44; Q_3_ 6:55) for patients with diabetes and 1 h, 15 min, (Q_1_ 0:31; Q_3_ 3:17) for patients without diabetes. The difference in median delay, 1 h, 09 min, was statistically significant (*p* = 0.024). Approximately 54 % of patients with diabetes delayed for 2 h or more compared with 36 % of patients without diabetes (*p* = 0.002), and 26 % of patients with diabetes and 16 % of patients without diabetes had delay times that exceeded 6 h (*p* =0.021) (Fig. 1). After adjustments for sex and age, patients with diabetes had higher risk for delay of ≥ 2 h, OR 2.04 (95 % CI: 1.26-3.30, *p* = 0.004).

## Discussion

In this multicentre study, we found that patients with diabetes had a considerable longer patient delay compared to patients without diabetes, with a difference of 1 h and 9 min between the groups. As many as half of patients with diabetes exceeded 2 h delay, making the goal of a total ischemic time under 2 h impossible [1]. Also after adjusting for age and sex, diabetes was significantly associated to a patient delay exceeding 2 h. The prolonged delay is consistent with a previous study based on The Northern Sweden MONICA myocardial infarction registry, which also found that diabetes was associated with pre-hospital delay for more than 2 h [27]. Furthermore, it has also been shown that patients with diabetes have delayed initiation of reperfusion treatments, leading to longer ischemic time [28, 29] contributing to their worse outcome in MI.

The reason for longer delays in diabetes is obscure and still debated. Diabetes is a well-known risk factor for development of ischemic heart disease and patients with diabetes ought to be informed by their health care providers about MI symptoms and warning signs, and thus be able to seek care more promptly than patients without diabetes. Instead the opposite is found in our as well as previous studies [13, 17]. In the present study some differences in symptom presentation were found, such as more shoulder pain/discomfort, shortness of breath, and tiredness in patients with diabetes - symptoms that may not be associated with MI and thus have caused a prolonged delay. This is in line with previous research that found that dyspnea [16, 18] and fatigue [30] were more common in patients with diabetes. Kentch et al. [18] suggest that the higher prevalence of dyspnea in patients with diabetes might be due to a more severe stage of coronary artery disease among these patients. This could also be a possible explanation in our study since patients with diabetes more often had previous angina pectoris and a history of MI. In the current study patients with diabetes were less likely to experience cold sweat, which might be due to diabetic autonomic neuropathy, in which loss of sweating can be one of the symptoms [31]. Tiredness can be difficult to evaluate, especially without concurrent chest pain, since such diffuse symptom can be present in many different diseases including diabetes [32, 33].

On the other hand chest pain/discomfort during MI was just as common in patients with diabetes and those without and there were no differences in pain intensity or number of symptoms. Similar findings of no differences in chest pain [18, 34] or pain intensity [18] are previously reported while other studies have found that patients with diabetes less frequently experience chest pain in MI [16, 35]. The latter are older than the present study, with data collected between 1990–1995 [16] and 1994–1998 [35] and this can contribute to the divergent results. Modern diabetic treatment and better glucose control may have decreased diabetes complications, such as cardiac autonomic neuropathy - a complication that can lead to absence of chest pain [36].

There are also other factors, such as symptom interpretation that may contribute to patient delay. Previous research has shown that the evaluation of symptoms in the presence of chronic illness might be difficult [37, 38]. A qualitative meta-analysis of heart failure self-care among people with multiple comorbid conditions reports that the patient’s perceptions of self-care might interact with fragmented information from different providers. This makes it difficult when patients are forced to make decisions about their symptoms; for example, whether the symptoms are related to their diabetes, their kidneys, or their heart [38]. Patients with diabetes in the present study often had concomitant hypertension and angina pectoris, and that might have affected their symptom interpretation and their decision to seek medical care.

In our study, only 67 % of patients interpreted their symptoms as cardiac in origin. This is somewhat surprising because over 80 % in both groups reported chest pain, and people often recognize chest pain as a symptom of MI [39]. It is possible that other factors such as intermittent symptoms confuse patients who might expect MI to have a sudden onset and persistent symptoms. In this study, about one quarter in both groups reported the symptoms to “come and go”. A previous study found that 65 % of patients with acute coronary syndrome experienced a slow onset presentation (described as any typical or atypical MI symptom with gradual onset, mild intensity, and intermittent nature) [40].

### Strengths and limitations

This study offers new insights about symptoms and the pre-hospital phase in patients with diabetes. The strength of this study is underscored by the inclusion of a large number of patients from five hospitals and from different areas of Sweden, adding to the external validity of our findings. Patients were included within 24 h after admission to hospital and the time limit was chosen to reduce the risk of recall bias. We applied no age restraints, therefore the generalizability should be high, at least for Swedish health care. Using a validated questionnaire covering the most important aspects of patient delay was supplemented by a thorough analysis of objectively noted time points in the medical journals, including those from ambulance transports. One limitation is that participants had to be pain free and hemodynamically stable before participating in the study. Some patients admitted during the study period were not stabilized within 24 h, and this might mean that the sickest patients were not included in the study. Another limitation is that the self-reported questionnaire is only available in Swedish, which might complicate comparisons with other studies. There is also a possibility that patients’ memories of their pre-hospital experiences were affected by analgesic and sedative drugs.

### Implications

The knowledge gained from our study is important not only for health care personnel meeting patients with a suspected MI, but also for the education of people with diabetes. Most patients with diabetes have regular contact with diabetes specialist nurses or doctors that allows them to have face-to-face discussions about symptoms and how to react if MI symptoms occur. Patient education needs to emphasize the possibility that MI could start with diffuse symptoms such as tiredness and shortness of breath and that MI could present as a slow onset event with intermittent symptoms. Knowledge about differences in symptoms may also be used to design interventions aiming to reduce prehospital delay in patients with diabetes. A reduction in patient delay time among patients with diabetes can improve survival since longer prehospital delay in patients with diabetes might contribute to their worse outcome in MI compared to patients without diabetes.

## Conclusions

Our study demonstrated that patients with diabetes had longer patient delay than patients without diabetes. Chest pain was just as common both in patients with and without diabetes, and there were no differences in the number of MI symptoms or symptom intensity. This study also showed that many patients with diabetes have other chronic illnesses such as hypertension and angina that might complicate their interpretation of symptoms. Further research is needed into how people with a chronic illness such as diabetes respond to new acute symptoms such as those of MI and how the health care system can support them in decision making despite their different diseases.

## Abbreviations

CI, confidence interval; EMS, emergency medical service; FMC, first medical contact; Md, median; MI, myocardial infarction; NRS, numeric rating scale; NSTEMI, non-ST-elevation myocardial infarction; OR, odds ratio; Q, quartiles; Sd, standard deviation; STEMI, ST-elevation myocardial infarction.

## References

[1] Boersma E, Maas AC, Deckers JW, Simoons ML (1996). Early thrombolytic treatment in acute myocardial infarction: reappraisal of the golden hour. Lancet.

[2] Koul S, Andell P, Martinsson A, Gustav Smith J, van der Pals J, Schersten F (2014). Delay from first medical contact to primary PCI and all-cause mortality: a nationwide study of patients with ST-elevation myocardial infarction. J Am Heart Assoc.

[3] Steg PG, James SK, Atar D, Badano LP, Lundqvist CB, Borger MA (2012). ESC Guidelines for the management of acute myocardial infarction in patients presenting with ST-segment elevation: The Task Force on the management of ST-segment elevation acute myocardial infarction of the European Society of Cardiology (ESC). Eur Heart J.

[4] Kaul P, Armstrong PW, Sookram S, Leung BK, Brass N, Welsh RC (2011). Temporal trends in patient and treatment delay among men and women presenting with ST-elevation myocardial infarction. Am Heart J.

[5] Moser DK, Kimble LP, Alberts MJ, Alonzo A, Croft JB, Dracup K (2006). Reducing delay in seeking treatment by patients with acute coronary syndrome and stroke: a scientific statement from the American Heart Association Council on cardiovascular nursing and stroke council. Circulation.

[6] Isaksson RM, Brulin C, Eliasson M, Naslund U, Zingmark K (2011). Prehospital experiences of older men with a first myocardial infarction: a qualitative analysis within the Northern Sweden MONICA Study. Scand J Caring Sci.

[7] Johansson I, Swahn E, Stromberg A (2007). Manageability, vulnerability and interaction: a qualitative analysis of acute myocardial infarction patients’ conceptions of the event. Eur J Cardiovasc Nurs.

[8] O’Donnell S, Moser DK (2012). Slow-onset myocardial infarction and its influence on help-seeking behaviors. J Cardiovasc Nurs.

[9] Angerud KH, Brulin C, Eliasson M, Naslund U, Hornsten A. The Process of Care-seeking for Myocardial Infarction Among Patients With Diabetes. J Cardiovasc Nurs. 2014. 10.1097/JCN.0000000000000195 PMC4540334 25325370

[10] Cameron L, Leventhal EA, Leventhal H (1993). Symptom representations and affect as determinants of care seeking in a community-dwelling, adult sample population. Health Psychol.

[11] Brink E, Karlson BW, Hallberg LR-M (2002). To be stricken with acute myocardial infarction: A grounded theory study of symptom perception and care-seeking behaviour. J Health Psychol.

[12] Khraim FM, Carey MG (2009). Predictors of pre-hospital delay among patients with acute myocardial infarction. Patient Educ Couns.

[13] Saczynski JS, Yarzebski J, Lessard D, Spencer FA, Gurwitz JH, Gore JM (2008). Trends in prehospital delay in patients with acute myocardial infarction (from the Worcester Heart Attack Study). Am J Cardiol.

[14] O’Donnell S, Condell S, Begley C, Fitzgerald T (2006). Prehospital care pathway delays: gender and myocardial infarction. J Adv Nurs.

[15] McGinn AP, Rosamond WD, Goff DC, Taylor HA, Miles JS, Chambless L (2005). Trends in prehospital delay time and use of emergency medical services for acute myocardial infarction: experience in 4 US communities from 1987-2000. Am Heart J.

[16] Culic V, Eterovic D, Miric D, Silic N (2002). Symptom presentation of acute myocardial infarction: influence of sex, age, and risk factors. Am Heart J.

[17] Ting HH, Bradley EH, Wang Y, Lichtman JH, Nallamothu BK, Sullivan MD (2008). Factors associated with longer time from symptom onset to hospital presentation for patients with ST-elevation myocardial infarction. Arch Intern Med.

[18] Kentsch M, Rodemerk U, Gitt AK, Schiele R, Wienbergen H, Schubert J (2003). Angina intensity is not different in diabetic and non-diabetic patients with acute myocardial infarction. Z Kardiol.

[19] Johansson I, Stromberg A, Swahn E (2004). Factors related to delay times in patients with suspected acute myocardial infarction. Heart Lung.

[20] Ryden L, Standl E, Bartnik M, Van den Berghe G, Betteridge J, de Boer MJ (2007). Guidelines on diabetes, pre-diabetes, and cardiovascular diseases: executive summary. The Task Force on Diabetes and Cardiovascular Diseases of the European Society of Cardiology (ESC) and of the European Association for the Study of Diabetes (EASD). Er Heart J.

[21] Eliasson M, Jansson JH, Lundblad D, Naslund U (2011). The disparity between long-term survival in patients with and without diabetes following a first myocardial infarction did not change between 1989 and 2006: an analysis of 6,776 patients in the Northern Sweden MONICA Study. Diabetologia.

[22] Norhammar A, Lindback J, Ryden L, Wallentin L, Stenestrand U (2007). Improved but still high short- and long-term mortality rates after myocardial infarction in patients with diabetes mellitus: a time-trend report from the Swedish Register of Information and Knowledge about Swedish Heart Intensive Care Admission. Heart (British Cardiac Society).

[23] Svensson AM, Dellborg M, Abrahamsson P, Karlsson T, Herlitz J, Duval SJ (2007). The influence of a history of diabetes on treatment and outcome in acute myocardial infarction, during two time periods and in two different countries. Int J Cardiol.

[24] Hamm CW, Bassand JP, Agewall S, Bax J, Boersma E, Bueno H (2011). ESC Guidelines for the management of acute coronary syndromes in patients presenting without persistent ST-segment elevation: The Task Force for the management of acute coronary syndromes (ACS) in patients presenting without persistent ST-segment elevation of the European Society of Cardiology (ESC). Eur Heart J.

[25] Thylen I, Ericsson M, Hellstrom Angerud K, Isaksson RM, Sederholm LS (2015). First medical contact in patients with STEMI and its impact on time to diagnosis; an explorative cross-sectional study. BMJ Open.

[26] World Medical Association I (2013). WMA Declaration of Helsinki - Ethical Principles for Medical Research Involving Human Subjects. JAMA.

[27] Angerud KH, Brulin C, Naslund U, Eliasson M (2013). Longer pre-hospital delay in first myocardial infarction among patients with diabetes: an analysis of 4266 patients in the northern Sweden MONICA Study. BMC Cardiovasc Disord.

[28] Windecker S, Kolh P, Alfonso F, Collet JP, Cremer J, Falk V (2014). 2014 ESC/EACTS Guidelines on myocardial revascularization: The Task Force on Myocardial Revascularization of the European Society of Cardiology (ESC) and the European Association for Cardio-Thoracic Surgery (EACTS) Developed with the special contribution of the European Association of Percutaneous Cardiovascular Interventions (EAPCI). Eur Heart J.

[29] Ryden L, Grant PJ, Anker SD, Berne C, Cosentino F, Danchin N (2013). ESC Guidelines on diabetes, pre-diabetes, and cardiovascular diseases developed in collaboration with the EASD: the Task Force on diabetes, pre-diabetes, and cardiovascular diseases of the European Society of Cardiology (ESC) and developed in collaboration with the European Association for the Study of Diabetes (EASD). Eur Heart J.

[30] DeVon HA, Penckofer S, Larimer K (2008). The association of diabetes and older age with the absence of chest pain during acute coronary syndromes. West J Nurs Res.

[31] Vinik AI, Freeman R, Erbas T (2003). Diabetic autonomic neuropathy. Semin Neurol.

[32] Fritschi C, Quinn L (2010). Fatigue in patients with diabetes: a review. J Psychosom Res.

[33] Segerstedt J, Lundqvist R, Eliasson M (2015). Patients with type 1 diabetes in Sweden experience more fatigue than the general population. J Clin Transl Endocrinol.

[34] Angerud KH, Brulin C, Naslund U, Eliasson M (2012). Patients with diabetes are not more likely to have atypical symptoms when seeking care of a first myocardial infarction. An analysis of 4028 patients in the Northern Sweden MONICA Study. Diabet Med.

[35] Canto JG, Shlipak MG, Rogers WJ, Malmgren JA, Frederick PD, Lambrew CT (2000). Prevalence, clinical characteristics, and mortality among patients with myocardial infarction presenting without chest pain. JAMA.

[36] Dimitropoulos G, Tahrani AA, Stevens MJ (2014). Cardiac autonomic neuropathy in patients with diabetes mellitus. World J Diabetes.

[37] Riegel B, Jaarsma T, Stromberg A (2012). A middle-range theory of self-care of chronic illness. ANS Advances Nurse Sci.

[38] Dickson VV, Buck H, Riegel B (2011). A qualitative meta-analysis of heart failure self-care practices among individuals with multiple comorbid conditions. J Card Fail.

[39] Goff DC, Mitchell P, Finnegan J, Pandey D, Bittner V, Feldman H (2004). Knowledge of heart attack symptoms in 20 US communities. Results from the Rapid Early Action for Coronary Treatment Community Trial. Prev Med.

[40] O’Donnell S, McKee G, Mooney M, O’Brien F, Moser DK (2014). Slow-onset and fast-onset symptom presentations in acute coronary syndrome (ACS): new perspectives on prehospital delay in patients with ACS. J Emerg Med.

